# Quantification of Age-Related Changes in the Lateral Organization of the Lipid Portion of the Intact Membranes Isolated from the Left and Right Eye Lenses of the Same Human Donor

**DOI:** 10.3390/membranes13020189

**Published:** 2023-02-03

**Authors:** Laxman Mainali, Marija Raguz, Witold Karol Subczynski

**Affiliations:** 1 Department of Physics, Boise State University, Boise, ID 83725, USA; 2 Biomolecular Sciences Graduate Program, Boise State University, Boise, ID 83725, USA; 3 Department of Medical Physics and Biophysics, University of Split School of Medicine, 21000 Split, Croatia; 4 Department of Biophysics, Medical College of Wisconsin, Milwaukee, WI 53226, USA

**Keywords:** eye lens, membrane domains, cholesterol, phospholipids, EPR, spin labeling

## Abstract

The continuous wave EPR spin-labeling method was used to evaluate age-related changes in the amounts of phospholipids (PLs) and cholesterol (Chol) in domains present in intact, cortical, and nuclear fiber cell plasma membranes isolated separately from the left and right eye lenses of the same human donor. The relative amounts of boundary plus trapped PLs were evaluated with the PL analog 12-doxylstearic acid spin label (12-SASL) and the relative amounts of trapped Chol with the Chol analog androstane spin label (ASL). The donors ranged in age from 15 to 70 years. Both the left and right eye lenses from donors aged 60, 65, and 70 years had nuclear cataracts; additionally, the right eye lens only of the 60-year-old donor had a cortical cataract. In transparent lenses, the relative amounts of boundary plus trapped PLs increase monotonously with donor age, and, at all ages, this amount was greater in nuclear compared with cortical membranes. Moreover, in transparent lenses, the relative amount of trapped Chol increases with age in nuclear membranes. However, the EPR spectrum of ASL from cortical membranes of 15- to 60-year-old donors shows only the weakly immobilized component assigned to ASL in the bulk plus Chol bilayer domain. Only the cortical membranes of 61- to 70-year-old donors contain both weakly and strongly immobilized components. The strongly immobilized component is assigned to ASL in trapped lipids. We speculate that the age of 60 years may be considered as a “threshold” for appearance of trapped lipids in cortical membranes. The relative amounts of boundary plus trapped PLs in lenses with nuclear cataracts is lower than that predicted from the tendency of the age-dependent increase observed for transparent lenses. The differences in amounts of lipids in the indicated left and right eye domains of each donor are smaller than the differences in single donors of a similar age.

## 1. Introduction

Over the last decade, our work has concentrated on discrimination and characterization of membrane domains in total lipids isolated from intact animal [1,2,3,4] and human [5,6,7] cortical and nuclear fiber cell membranes in the eye lens, as well as discrimination of domains in intact animal [8] and human [9,10,11] fiber cell membranes of the eye lens. Properties of the latter were characterized in detail using state-of-the-art electron paramagnetic resonance (EPR) techniques and spin-labeling methods developed at the National Biomedical EPR Center, Medical College of Wisconsin. First, these techniques and methods enabled us to work with a pool of approximately 20 cortical and nuclear eye lens samples from animals [8] and humans [9,10]. Use of many samples enabled us to develop methods to evaluate transmembrane profiles of basic membrane properties, including order, fluidity, oxygen transport parameter, and hydrophobicity. The significance of these data is summarized in reviews [12,13,14]. Next, these techniques and methods were advanced to a level that enables measurements on lenses from a single donor, i.e., two lenses, thus permitting consideration of the health history of the donor in the data analysis [10]. Finally, these technologies and methods have been further advanced to enable measurements on intact, cortical, and nuclear fiber cell plasma membranes isolated separately from the left and right eye lenses of the same donor [10,15]. Use of the same donor provides the opportunity to study the fiber cell membrane properties of donors with the same basic health histories but with different health histories for individual eye lenses. Nuclear and/or cortical cataracts can develop in one lens while the second lens remains transparent. Drops decreasing eye internal pressure are frequently administered only to one eye. Additionally, different eye diseases, such as glaucoma and age-related macular degeneration, which can develop in one eye, can be included in data analysis.

One direction of our research was to develop EPR spin-labeling methods to quantify lipids in the domains of intact biological membranes, in this case domains of fiber cell plasma membranes of the eye lens. We identified four distinct lipid domains in intact fiber cell plasma membranes: the bulk lipid domain, the boundary lipid domain, the trapped lipid domain, and the cholesterol bilayer domain (CBD). The bulk lipid domain in the intact membrane is formed by the lipid bilayer, which is unaffected by the presence of integral membrane proteins and has the same (or almost the same) properties as a lipid bilayer membrane formed from the whole lipids extracted from the intact membrane. The boundary lipid domain around integral proteins is formed by immobilized phospholipids bounded to proteins (a single lipid layer coating the hydrophobic surface of the protein regardless of the amount of fluid bulk lipids) [16]. The exchange rate between boundary and bulk lipids is of the order of magnitude of 10^7^ s^−1^ or greater [17,18,19]. The trapped lipid domain, also called the slow oxygen transport (SLOT) domain, is formed by lipids in contact with two proteins and/or by lipids in contact with proteins and boundary lipids [20]. The exchange rate of lipids between this domain and the bulk plus boundary regions is of the order of magnitude of 10^5^ s^−1^ or smaller [21]. The acyl chains are very immobile in the trapped lipid domain, and membrane dynamics are suppressed to the level of the gel-phase. The CBD domain is a transmembrane pure cholesterol bilayer immersed in the bulk phospholipid/cholesterol membrane [22,23,24]. The presence of CBDs ensures that the surrounding PL bilayer is saturated with Chol at a Chol/PL ratio of one, forming a structured [25] or dispersed [26] liquid-ordered phase.

**Figure 1:**
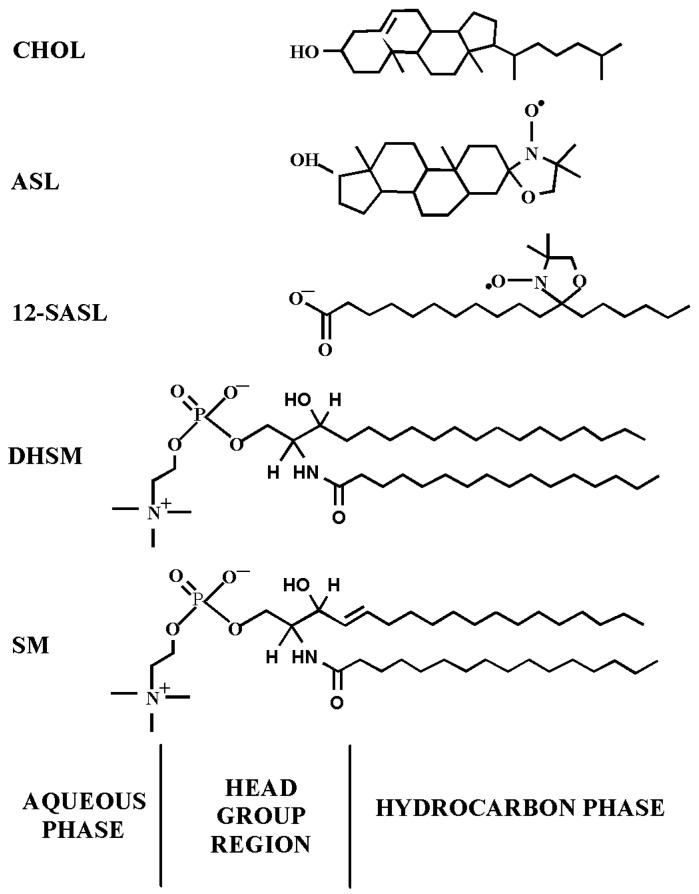
Chemical structures of spin labels used and their approximate locations in the lipid bilayer membrane. The PL analog 12-SASL and the Chol analog ASL, respectively, model the properties of the membrane PLs acyl chain region and the behavior of Chol molecules in the lipid bilayer. Chemical structures of palmitoyl sphingomyelin (SM) and palmitoyl dihydrosphingomyelin (DHSM), the most abundant lipids in human lens membranes [40], are also presented.

We developed two methodological approaches that enabled respective evaluation of amount of phospholipids (PLs), as a mol% of total PLs, with use of PL analog 12-doxylstearic acid spin label (12-SASL) and amount of cholesterol (Chol), as a mol% of total Chol, with use of Chol analog androstane spin label (ASL) in membrane domains. See Figure 1 for spin-label structures and their approximate locations in the lipid bilayer. One methodological approach is based on analysis of the conventional continuous wave (CW) EPR spectra of spin labels [9,10,15], and the other is based on analysis of saturation recovery (SR) EPR signals of spin labels in deoxygenated samples [27]. The former approach enables evaluation of the relative amounts of boundary plus trapped phospholipids and the relative amounts of cholesterol in the trapped lipid domain. The latter approach enables evaluation of the relative amounts of PLs and Chol in the trapped lipid domain of intact membranes. Thus, both methods complement each other.

In the presented research, we used the CW EPR spin-labeling method to evaluate age-related changes in the amounts of PLs and Chol in domains of intact, cortical, and nuclear fiber cell plasma membranes isolated separately from the left and right eye lenses of the same donor. This improved method for quantification of lipid domains in intact fiber cell membranes permits detailed examination of differences due to aging and cataract formation. However, the CW EPR methods applied here, and also other methods we have developed, are not able to discriminate between cytoplasmic and extracellular leaflets of the membrane. We obtain the averaged information on membrane domain properties from both leaflets.

The long-term goal is to understand the mechanisms responsible for age-related nuclear cataract development as the responsible mechanisms cannot be discussed without understanding the changes that occur in normal transparent lenses with age [28,29,30,31]. Most likely, nuclear cataracts become apparent only after age-related changes in transparent lenses accumulate to a certain level. This may be the turning point after which cataract development is unavoidable.

Organization of lipids into domains strongly depends on organization of integral membrane proteins into arrays and aggregates, which, in turn, depends on interaction with the cytoskeleton [32,33,34,35,36,37,38,39]. Thus, we believe that detailed quantification of age-related changes in lateral organization of lipids will also provide information regarding organization of integral membrane proteins. Based on our data, we speculate that, in the cortical membranes of transparent lenses of human donors up to the age of 60 years, integral membrane proteins are organized to minimize amount of trapped lipids.

## 2. Materials and Methods

### 2.1. Materials

The cholesterol analog spin label (ASL) and doxylstearic acid spin label (12-SASL) were purchased from Molecular Probes (Eugene, OR, USA). Other chemicals of at least reagent grade were purchased from Sigma Aldrich (St. Louis, MO, USA).

### 2.2. Isolation of Intact Cortical and Nuclear Membranes from Human Lens

**Figure 2:**
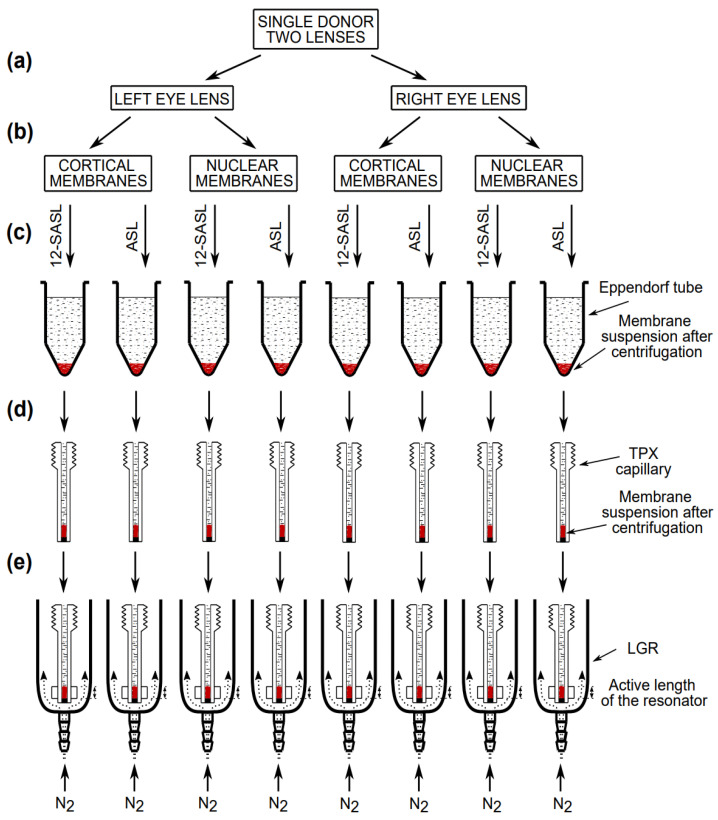
Schematic drawing showing the handling of samples with a small amount of a biological material for EPR measurements. (**a**) The left and right eye lenses from the same donor were separated for further membrane preparations. (**b**) Cortical and nuclear membranes were isolated as described in [8,10,15]. (**c**) One-half of each membrane suspension was added to a test tube, the bottom of which contained the dry film of 12-SASL or ASL. The test tube was shaken for about 2 h at room temperature, and the diluted, spin-labeled membrane suspension was transferred to an Eppendorf tube and concentrated by centrifugation to the volume of a TPX capillary. (**d**) The membrane suspension was further concentrated in the TPX capillary to match the active length of the resonator. (**e**) The TPX capillary was positioned inside the loop-gap resonator with the sample located exactly in the active length of the resonator. In the resonator, the sample was thoroughly deoxygenated by a stream of nitrogen. For more details, see [41].

Nine pairs of human lenses from donors of different ages, i.e., 15-year-old (female, white/Caucasian), 21-year-old (male, white/Caucasian), 36-year-old (male, white/Caucasian), 40-year-old (male, white/Caucasian), 46-year-old (male, white/Caucasian), 53-year-old (female, Black), 60-year-old (male, white/Caucasian), 65-year-old (male, white/Caucasian), and 70-year-old (male, white/Caucasian), were obtained from the Lions Eye Bank of Wisconsin. Lenses were removed in situ from bodies within an average time frame of 9 h postmortem. Human lenses were stored at −80 °C until intact, cortical, and nuclear membranes could be isolated. A binocular microscope was used to examine the lenses, and presence and absence of cataractous changes were evaluated using a subjective grading system based on the opacity of the lens for the cortical cataracts and amount of “yellowness” of the lens for the nuclear cataracts. The nuclear cataracts were detected in both the left and right eyes of the 60-, 65-, and 70-year-old donors, and a cortical cataract also was detected in the right eye of the 60-year-old donor. All other lenses were free of cataracts. The intact, cortical, and nuclear membranes from single human lenses were isolated and stored at −20 °C as described previously [8,9,10,15]. The separation of the cortex from the nucleus is presented schematically in Figure 2a,b.

### 2.3. EPR Measurements

As described previously [8,9,10,15], the membrane suspensions were spin-labeled with ASL and 12-SASL spin labels and then transferred to Eppendorf tubes, centrifuged (see Figure 2c), and the loose pellet was transferred to a 0.6 mm inner diameter capillary made of gas-permeable methylpentene polymer (TPX) (see Figure 2d). The TPX capillary with dense membrane suspension at the bottom was positioned in the loop-gap resonator for CW EPR measurements (Figure 2e). All EPR spectra for intact membranes were recorded at 37 °C with microwave power of 5.0 mW and modulation amplitude of 1.0 G. All samples were deoxygenated to measure the correct EPR lineshape. The EPR spectra for lens lipid membrane were measured at an appropriate temperature to match the spectral components in the EPR spectrum, as discussed in Section 2.4.

### 2.4. Method of Evaluating Relative Amounts of PL and Chol in Domains of Intact Membranes

**Figure 3:**
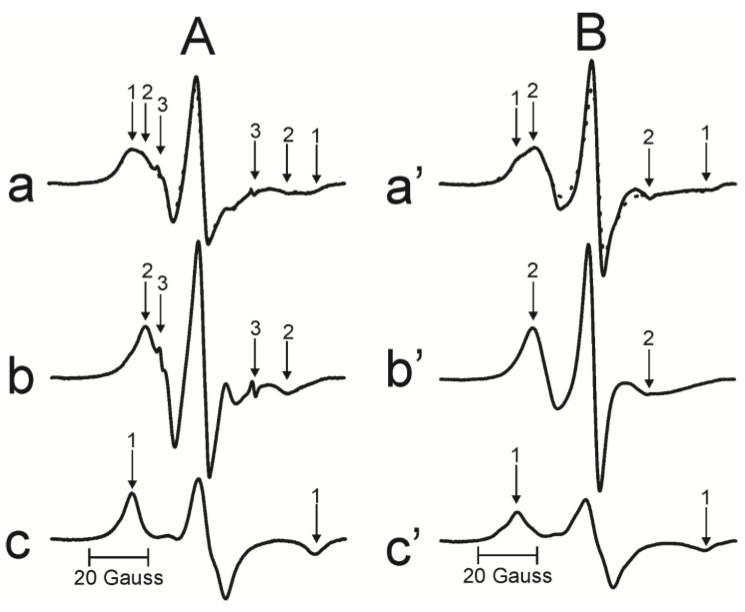
(**A**) The procedure for evaluating the relative amounts of PLs in domains in the lipid bilayer portion of intact membranes is illustrated based on the representative EPR spectrum of 12-SASL in nuclear membranes from the transparent left eye lens of a 46-year-old human donor obtained at 37 °C ((**a**)-solid line). Arrows 1–3 represent spectra from strongly immobilized, weakly immobilized, and water components, respectively. The EPR spectrum of nuclear LLM obtained with 12-SASL at 37 °C (**b**). The EPR spectrum of the nuclear LLM obtained with 12-SASL at −18 °C (**c**) (i.e., this spectrum has the same maximum splitting as the strongly immobilized component in spectrum (a-component 1)). The dotted spectrum ((**a**)-dotted) was obtained by adding 55% of spectrum (**b**) and 45% of spectrum (**c**). (B) The procedure for evaluating the relative amount of Chol in domains in the lipid bilayer portion of intact membrane is illustrated based on the representative EPR spectrum of ASL in nuclear membranes from the cataractous left eye lens of a 65-year-old human donor obtained at 37 °C ((**a’**)-solid line). Arrows 1 and 2 represent spectra from strongly immobilized and weakly immobilized components, respectively. The EPR spectrum of nuclear LLM obtained with ASL at 34 °C (**b’**). The EPR spectrum of nuclear LLM with ASL obtained at −58 °C (**c’**) (i.e., this spectrum has the same maximum splitting as the strongly immobilized component in spectrum (**a’**-component 1)). The dotted spectrum (**a’**-dotted line) was obtained by adding 50% of spectrum (**b’**) and 50% of spectrum (**c’**).

The relative amounts of PLs and Chol in domains of intact cortical and nuclear membranes derived from a single human lens were estimated as previously described in [15]. Briefly, the PL analog spin label, 12-SASL, is the best spin label to show the distribution of PLs in intact membranes because its EPR spectrum (Figure 3A(a)) shows clear separation between weakly and strongly immobilized components. The former is coming from 12-SASL located in the bulk lipid domain and the latter from 12-SASL located in the boundary and trapped lipid domains. The spectrum coming from intact membranes can be deconvoluted to determine the contribution of each component. Representative EPR spectra of 12-SASL (Figure 3A(a–c)) show the procedure for deconvolution of the EPR spectrum of 12-SASL obtained at 37 °C from the intact nuclear membranes derived from the left eye of the transparent human lens of a 46-year-old donor. The weakly immobilized component can be approximated by the EPR spectrum of 12-SASL in the nuclear lens lipid membrane (LLM), made of the total lipid extract from the nucleus, obtained at 37 °C (Figure 3A(b)). The strongly immobilized component can be approximated by the EPR spectrum of 12-SASL from the nuclear LLM obtained at −18 °C (Figure 3c). The maximum splitting in this spectrum is the same as the maximum splitting of the strongly immobilized component coming from intact membranes. It should be indicated that the maximum splitting is directly related to the order parameter of acyl chains [42]. To evaluate the relative distribution of PLs between the bulk and boundary plus trapped lipid domains, we have to be sure that spectra (b) and (c) were obtained for the same spin label content in the sample (the same number of spins), which was confirmed by the same areas under the absorption curves. With all these precautions, the EPR spectrum of 12-SASL from an intact nuclear membrane (a—solid line) can be reproduced by adding 55% of spectrum (b) from 12-SASL in bulk lipids and 45% of spectrum (c) from 12-SASL in boundary and trapped lipids. The fitted spectrum is shown as a dotted line in (a).

Representative EPR spectra of ASL (Figure 3B(a’–c’)) show the procedure for deconvolution of the EPR spectrum of ASL obtained at 37 °C from the intact nuclear membranes derived from the left eye of the cataractous human lens of a 65-year-old donor. The weakly and strongly immobilized components coming from ASL in bulk plus CBD lipids and the trapped lipids, respectively, are clearly separated. The weakly immobilized component can be approximated by the EPR spectrum of ASL in the nuclear LLM obtained at 34 °C (Figure 3B(b’)). The strongly immobilized component can be approximated by the EPR spectrum of ASL from the nuclear LLM by reducing the motion of ASL to that in the trapped lipid domain, which is achieved by decreasing the temperature for nuclear LLM to −58 °C (Figure 3B(c’)). To evaluate the relative distribution of Chol molecules between the bulk plus CBD and the trapped lipid domains, care was taken to obtain spectra (b’) and (c’) for the same spin label content in the sample, which was confirmed by the same areas under the absorption curves. With all these precautions, the EPR spectrum of ASL from an intact nuclear membrane (a’—solid line) can be reproduced by adding 50% of spectrum (b’) from ASL in bulk lipids plus CBDs and 50% of spectrum (c’) from ASL in trapped lipids. The fitted spectrum is shown as a dotted line in (a’).

Final results are expressed as mol% of total PLs and Chol. The precision of the evaluations of the boundary plus trapped PLs using 12-SASL and the trapped Chol using ASL was better than 5% because a 5% change in the contribution of each component decreased the goodness of fit (see Section 2.5 for evaluation of sample-to-sample variability). Here, we applied these procedures to intact cortical and nuclear membranes isolated separately from the left and right eye lenses of nine human donors of different ages.

### 2.5. Statistical Analysis (Sample-To-Sample Variability)

To evaluate of the age- and cataract-related differences between single human lenses, we must clearly determine which changes are due to differences between lenses and which are due to data scattering-related preparation/technique factors. This task is not easy because the properties of human lenses depend not only on the age of the donor but on many factors connected with the donor’s health history. This health history can be significantly different even for the left and right eye lenses. Our published paper describes in detail how we solved this problem (see Section 3.4 in [15]); here, we reiterate basic points and conclusions that are significant to our current investigations. The sample-to-sample preparation/technique-related changes were evaluated for cortical and nuclear lens membranes prepared from single porcine eye lenses from two-year-old animals that were grown in the same conditions. Thus, we assumed that differences between samples are mainly due to preparation/technique-related changes. From these measurements, we established that, in our future analyses, differences of 3.5% and 3.4% in amounts of PLs in intact, cortical, and nuclear human membranes, respectively, will be considered as statistically significant, with *p* ≤ 0.05. For differences in the amounts of Chol in intact, cortical, and nuclear human membranes, these values will be 4.7% and 3.7%, respectively. Values that are equal to or greater than the indicated values will not be considered significant due to preparation/technique factors.

## 3. Results

### 3.1. Amounts of PLs in Membrane Domains

**Figure 4:**
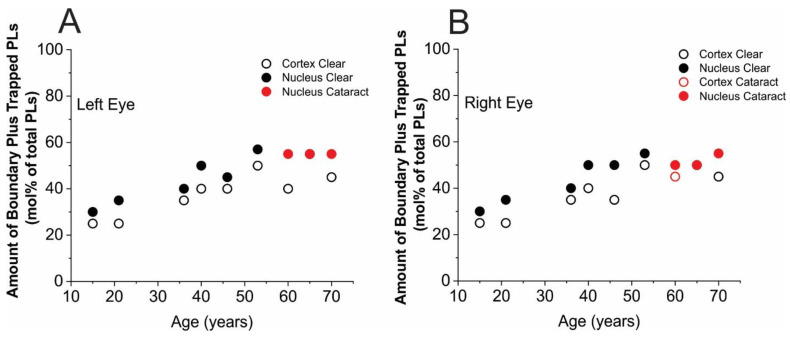
Amounts of boundary plus trapped PLs (mol% of total PLs in domains uniquely formed due to the presence of membrane proteins) in single, intact, human cortical (○), and nuclear (●) lens membranes. Data obtained from left eye lenses (**A**) and right eye lenses (**B**) are included. Both the left and right eyes of donors aged 60, 65, and 70 years have nuclear cataracts (●) and only the right eye of the 60-year-old donor has a cortical cataract (○).

Our data include nine left and nine right eye lenses from the nine donors. These data include six transparent lenses and three cataractous lenses, all of which have nuclear cataracts and one of which also has a cortical cataract. All the cumulative results indicating the amount of PLs in boundary plus trapped domains (mol% of total PLs) are presented eparately for left (Figure 4A) and right (Figure 4B) eye lenses. A few trends can be pointed out: (1) in transparent lenses, the amount of PLs in boundary plus trapped lipid domains steadily increases with age of donor, up to ~53 years, for nuclear and cortical membranes. After that age, both left and right eye lenses obtained from the eye bank demonstrate presence of nuclear cataracts. Additionally, a cortical cataract was detected in the right eye lens of a 60-year-old donor. Including cataractous lenses, the age-related increase levels off after the age of 53 years. Data scattering from the indicated trends is small for nuclear membranes and very large for cortical membranes in donors aged 40 years or greater. (2) For cataractous lenses, the amount of PLs in boundary plus trapped lipid domains of nuclear membranes is greater than that in cortical membranes for donors of all ages. Keeping in mind the statistical analysis described in Section 2.5, these differences are statistically significant and are not due to preparation/technique factors. (3) Overall, we can tell that the differences between the left and right eye lenses, considering cortical and nuclear membranes separately, are smaller than the differences between lenses from similarly aged donors. For some samples, the differences are even smaller than the preparation/technique limits. Interestingly, the data obtained a few years earlier for pooled lenses obeyed the indicated trends [9,10]. We should note here that the data are presented as a percent of boundary plus trapped lipids from the total PLs in membranes. Thus, the remaining amounts of PLs to 100% indicate the amount of PLs in the bulk domain.

### 3.2. Amounts of Chol in Membrane Domains

**Figure 5:**
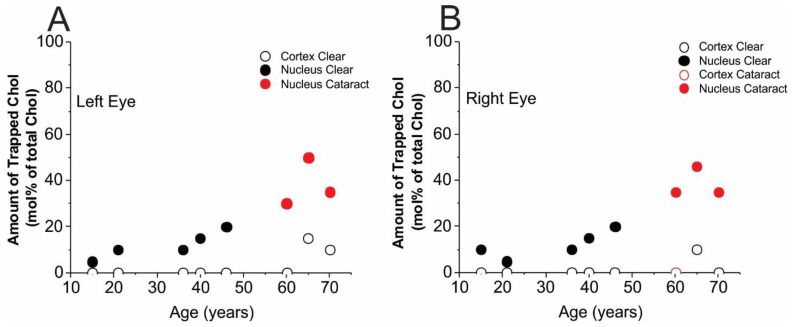
Amounts of trapped Chol (mol% of total Chol in domains uniquely formed due to the presence of membrane proteins) in single, intact, human cortical (○), and nuclear (●) lens membranes. Data obtained from the left eye lenses (**A**) and right eye lenses (**B**) are included. Both the left and right eyes of donors aged 60, 65, and 70 years have nuclear cataracts (●) and only the right eye of the 60-year-old donor has a cortical cataract (○).

Cumulative results for the trapped Chol are presented separately for the left (Figure 5A) and right (Figure 5B) eye lenses. As follows from Section 2.4, the method enabled evaluation of the amounts of Chol in trapped lipids as a mol% of total membrane Chol (in bulk plus CBDs and in trapped lipids). Keeping in mind that Chol molecules, as well as ASL molecules, are substantially excluded from boundary lipid domains, we can conclude that the remaining amounts of Chol to 100% indicate the amount of Chol in the bulk domain and in CBDs. Here, we also want to indicate basic trends in changes in amount of trapped Chol that occur with age of donor between cortical and nuclear membranes and between the left and right eyes: (1) the most amazing conclusion from the results presented in Figure 5 is that, up to the age of ~60 years, no trapped Chol was detected in the cortical membranes of either the left or right eye lenses. After the age of 60 years, a small amount of trapped Chol, around 10 mol% of total Chol, was detected in transparent lenses. We speculate that the age of 60 years may be considered as a “threshold” for appearance of trapped lipids in cortical membranes. A similar distribution of Chol in cortical lens membranes was obtained for pooled human cortical lenses [10]. (2) In nuclear membranes, Chol in trapped lipid domains was observed for donors of all ages and the amount of trapped Chol was observed to increase with the age of the donor. However, because of data scattering, it is difficult to make more certain conclusions. Certainly, that amount increases from 10–20 mol% for donors aged 15 to 20 years and to 40–50 mol% for donors aged 55 to 70 years. It also follows that amount of Chol in trapped lipid domains of nuclear membranes is greater than that in cortical membranes for donors of all ages and for cataractous lenses. (3) For Chol distribution between domains, we can tell that the differences between the left and right eye lenses (considering cortical and nuclear membranes separately) are smaller than differences between lenses from similarly aged donors, and these differences are statistically significant. Moreover, the data obtained a few years earlier for pooled lenses obeyed the indicated trends [9,10].

## 4. Discussion

**Figure 6:**
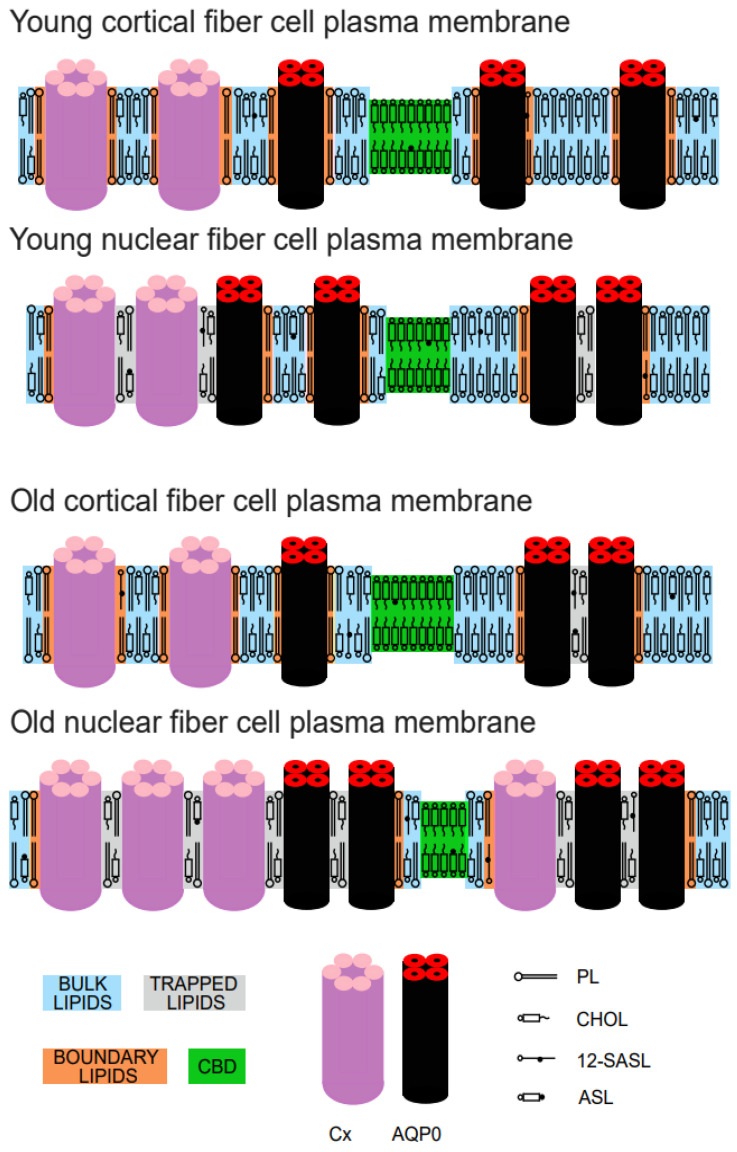
Schematic drawing showing the changes in the organization of lipids and proteins in intact, human eye lens fiber cell plasma membranes between cortical and nuclear membranes from young donors (between 0 and 20 years old) and between cortical and nuclear membranes from old donors (between 60 and 70 years old). Purported lipid domains induced by the presence of integral membrane proteins (mainly connexins and aquaporins) and high Chol content are indicated. The cytoskeletal and peripheral membrane proteins are not shown in the schematic. The PL spin label 12-SASL is distributed between bulk lipids, boundary lipids, and trapped lipids, while the Chol analog ASL is distributed between bulk lipids, trapped lipids, and CBD. Note that Chol is excluded from boundary lipids. The nitroxide moiety of 12-SASL and ASL is indicated by a black dot.

Our results allow us to conclude that the data scattering obtained for the amount of boundary and trapped PLs in nuclear membranes, both in the left and right eye (Figure 4), is very small and obeys the increasing trend (Figure 6A in Ref. [10]). However, the data scattering for Chol is much larger (e.g., compare Figure 5 with Figure 6B in [10]). Both old and new data show that ASL did not detect trapped lipids in cortical membranes in donors up to the age of ~60 years; however, in nuclear membranes, this domain is clearly detected by ASL (see Figure 5 and Figure 6B in [10]). In cortical membranes, small amounts of trapped lipids, indicated by ASL, were detected only for donors aged 60 years or greater; thus, we can conclude that the age of 60 years is a “threshold” for appearance of trapped lipids in cortical membranes. These major tendencies in lipid changes in domains in intact, cortical, and nuclear membranes are schematically illustrated in Figure 6 for young, between 0 and 20 years old, and old, between 60 and 70 years old, donors. From the ASL data (Figure 5), we can see that that integral membrane proteins are organized such that the cortical membranes of younger persons contain minimal amounts of trapped lipids, which appear only in older individuals. Because 12-SASL indicated significant amounts of boundary plus trapped lipids in the cortical membranes of donors of all ages, we can conclude that integral membrane proteins are inserted into the lipid bilayer of cortical membranes as a single (monomeric) protein or form functional aggregates without creating trapped lipid domains (as tetramers of aquaporins or complexes of six connexins molecules, which are only surrounded by boundary lipids). In nuclear membranes, ASL and 12-SASL indicated the presence of both domains induced by the presence of integral membrane proteins, namely boundary and trapped lipids. The amount of lipids in these domains increases with donor age.

We think it is useful to add here the information on how the unique lipid composition of human fiber cell membranes, containing high contents of saturated sphingolipids [43,44,45] and only traces of polyunsaturated fatty acids [46,47], may contribute to lipid–protein interactions and formation of boundary and trapped domains. Flexible saturated acyl chains can fit easier to the hydrophobic protein surface than unsaturated alkyl chains with a *cis*-double bond, which cause a 30° bend at this bond. The high degree of saturation ensures that the dynamics of alkyl chains decrease to the level of gel-phase membranes and do not decrease acyl chain order. With this explanation, our results do not contradict those presented by Sato et al. [48] and Zhang et al. [49], who showed that the structural order determined by the static measure of the trans/gauche rotamer ratio in the hydrocarbon chains of bovine and human lens membranes is not affected by the presence of intrinsic lens proteins.

In our previous papers [8,9,10,11] and in the beginning of the present publication, we focused our discussion on directly measured amounts of lipids in boundary and trapped lipid domains. However, data were obtained as relative amounts of boundary plus trapped PLs (mol% of total PLs) and as relative amounts of trapped Chol (mol% of total Chol). Thus, the remaining amounts to 100% indicate the amount of PLs in the bulk domain and the amount of Chol in the bulk domain and CBDs, all expressed in mol% of total PLs or Chol. Here, we would like to discuss our results more broadly, including all domains. Data obtained with ASL (Section 3.2 and Figure 5 and [9,10]) indicate that, in cortical membranes from transparent lenses (in donors up to the age of 60 years), all Chol molecules are in bulk domains plus CBDs. This result is significant as it shows that the trapped lipid domain is not detected (i.e., does not exist) in cortical membranes up to this age. This conclusion is strengthened by the fact that Chol and ASL molecules are substantially excluded from the boundary lipid domain [50,51,52,53,54,55]. We can broaden this conclusion to PLs, stating that, in these cortical membranes, PL molecules are located only in boundary lipids and in bulk lipids (~25 mol% in boundary domain and ~75 mol% in bulk lipid domain).

There is a constraint that is valid for all human fiber cell membranes, including cortical and nuclear membranes. Because these membranes are loaded with Chol [5,6] and CBDs are already present at young ages [7], the bulk domain is always saturated with Chol, with a Chol/PL molar ratio of 1/1, i.e., containing the same number of Chol and PL molecules. This indicated constraint established quantitative relations between the distributions of numbers of Chol and PL molecules in bulk domains in each membrane (cortical (young donors), cortical (old donors), nuclear (young donors), and nuclear transparent and cataractous (old donors)), which can differ from ratios of Chol and PL mol% established separately for total Chol and total PLs in each membrane.

For old cortical and nuclear membranes for which Chol content reaches the Chol solubility threshold of Chol/PL = 2/1 [6,7], another constraint is valid. Namely, at this condition, the number of Chol molecules in the bulk domain is equal to the number of Chol molecules in CBDs; thus, the number of Chol molecules in these two domains is two times greater than the number of PL molecules in the bulk domain. We would like to indicate once more that number of molecules in membrane domains, considering the constraints for distributions of Chol and PLs in membrane domains, is not the same as mol% of the distribution measured separately for Chol and PLs. The numbers must obey the measured distributions and constraints for each indicated membrane domain and domains in each membrane. To clearly demonstrate these relationships, we constructed Table 1 based on our obtained results and the indicated constraints. Table 1 summarizes our results for numbers of lipid molecules in membrane domains of transparent and cataractous lenses and the complementary schematic drawings in Figure 6. In the table, we present quantification data for all membrane domains (bulk, CBD, boundary, and trapped lipids). We focused on the data for cortical and nuclear membranes of transparent lenses of young (0 to 20 years old) and old (60 to 80 years old) donors for which the indicated constraints are valid. We also included data for membranes of 60- to 70-year-old donors with nuclear cataracts. The table presents an average of our new data presented here and the data we obtained previously [9,10]. The indicated constraints allow us to evaluate content (connections and ratios) in terms of the number of Chol and PL molecules in certain domains of each membrane. To better understand the distributions of Chol and PL molecules for the same domains (expressed as a molar ratio of Chol/PL and thus the ratio of numbers of molecules), we arbitrarily assigned a total number for PL molecules in all domains in each membrane of 100. These 100 molecules are distributed between domains in membranes according to the results obtained (mol% of total PLs). Of course, this assigned number can be different and can differ for each membrane. This assigned number enabled us to calculate and evaluate the number of Chol molecules in membrane domains as a ratio to 100 PL molecules (or, more specifically, as a ratio to the number of all PL molecules in the membrane). The calculated total number of Chol molecules can be smaller or greater than 100. Our evaluations of number of molecules are valid for each membrane only, and a comparison of numbers between membranes cannot be made. However, comparisons of ratios of molecules between domains and—thanks to this approach—between membranes in these particular domains obtained are certainly valid.

The relationships of Chol/PL in the bulk domain (at saturation limit) and in the bulk plus CBD (at solubility threshold) were based on investigation of model membranes and LLMs. However, in intact membranes, because of the presence of integral membrane proteins, several Chol and PL molecules are taken into boundary and trapped lipid domains. As stated in our previous papers [8,9,10,15], bulk lipid domains and bulk lipids plus CBD domains are minimally affected by presence of integral membrane proteins. This knowledge enabled us to add the indicated constraints on the distribution of Chol and PLs in the bulk domain at a Chol saturation limit of Chol/PL = 1/1 and a Chol distribution between the bulk domain and CBD at a Chol solubility threshold of Chol/PL = 2/1; it also enabled us to evaluate the number of Chol molecules in the bulk domain and CBD. Distributions of Chol molecules (mol% of total Chol) enabled us to obtain the number of Chol molecules in trapped lipid domains but only when the constraint “=” is valid in cortical and nuclear membranes of old donors. These numbers are included in Table 1. Using these data, we can estimate a molar ratio of Chol/PL in trapped lipid domains. We have exact numbers for Chol molecules in this domain; however, the evaluated number of PL molecules indicates the sum of PLs in boundary and trapped lipid domains. Therefore, we can evaluate the lower limits for the Cho/PL molar ratio in this domain and write that Chol/PL is 0, >0.2, >1.3, and >1.35 in the trapped lipid domain in cortical transparent (young donors’), cortical transparent (old donors’), nuclear transparent (old donors’), and nuclear cataractous (old donors’) fiber cell membranes. We can see that the ratio increases with age and depth in the lens (i.e., the depth is greater in nucleus than in cortex). Moreover, this ratio is not different for nuclear membranes from transparent and cataractous lenses.

The results presented in Table 1, when compared with data from the literature, indicate that organization of membrane proteins and organization of the lipid bilayer portion of fiber–cell membranes are closely related. The most abundant transmembrane integral proteins in human fiber cell membranes [33], aquaporin-0 (AQP0), and connexins (Cx46 and Cx50) [56,57] most likely induce formation of boundary and trapped lipids. AQP0 controls transport of water and some neutral solutes but not ions [58]. Connexins form gap junctions between lens fiber cells [59]. Cx46 is located mainly in the cortex and outer nuclear layers, and Cx50 is mainly in the nuclear core [60,61,62]. Gap junctions form ordered two-dimensional arrays in fiber cell membranes [34,35,36,37,38,39], and lipids can be trapped within these protein-rich structures. Density/distribution of these proteins varies as a function of fiber depth (age) [32]. The ordered arrays of AQP0 are enriched in the nucleus [36]. This is consistent with our current data showing that the amount of trapped lipids is greater in the nucleus than in the cortex.

As shown by Biswas et al. [63,64,65], significant structural remodeling of gap junctions occurs during fiber cell development and maturation. Gap junctions are selectively localized within the specialized interlocking membrane domains between lens fibers, called “ball-and-sockets” [64]. The newly formed gap junction channels in the superficial young fiber cells are constantly clustered and form large Chol-rich loosely packed gap junction plagues. In the mature inner cortical fibers, they are transformed into Chol-free tightly packed clusters [63,65]. This explains to some extent why in cortical membranes the relative amount of Chol trapped between membrane proteins is small and cannot be detected by conventional EPR. In nuclear membranes, which are enriched in ordered arrays of AQP0 [36], the amount of trapped lipids, including trapped Chol, is greater.

The experimental results enabled us to separately evaluate the relative distributions of Chol and PL molecules between the domains in each membrane. Sometimes, this amount is established as a sum for two domains and cannot be separated. The approach presented in Table 1 in some cases enables us to separate these distributions and obtain ratios of the number of Chol/PL in certain domains. Although the numbers of Chol and PL molecules in domains in different membranes cannot be compared, the ratios of these numbers can be compared. Thus, this new approach strengthens the results obtained experimentally and broadens the conclusions that can be made.

## 5. Conclusions

Our data were obtained on samples from the left and right eye lenses of donors of different ages, sexes, and races. Our data also include samples from three donors with cataractous lenses. We did not observe any major deviation from the indicated tendencies for donors of different sexes or races. Moreover, the differences between the left and right eye lenses are smaller than the differences between the lenses from donors of similar ages. Although the data scattering obtained for old donors is large, we can see the tendency of lenses with nuclear cataracts to have lower amounts of boundary and trapped lipids. However, the ratio of Chol/PL in these lenses does not differ when compared with age-matched transparent lenses. Because of the limited number of lenses, especially cataractous lenses, we can indicate only the major age-related changes in transparent lenses and some tendencies observed for lenses with nuclear cataracts.

We should state that the delicate relationship between lipid and integral protein content as well as solubilities of lipid molecules (i.e., Chol and PLs) in membrane lipid domains (according to solubility constants and solubility thresholds) affect organization of both lipids and integral proteins in membranes. In our work, we evaluated these relationships, which connect to organization of lipids and proteins. We believe that advancements in the EPR technique and developments of new spin labeling methods will enable us to obtain more detailed information on this subject.

## Figures and Tables

**Table 1:** Relative amounts of Chol and PLs in domains of intact, cortical, and nuclear fiber cell plasma membranes (expressed as mol% of total Chol or total PLs) from transparent lenses of young (0 to 20 years old) and old (60 to 80 years old) donors and in membranes from lenses with nuclear cataracts from 60- to 70-year-old donors. The numbers of Chol and PL molecules in these domains are evaluated considering the constraints connecting the numbers of Chol and PL molecules in certain domains. Data for mol%s are averaged from the present paper and our previous publications [9,10]. Only the ages of the donors are considered.

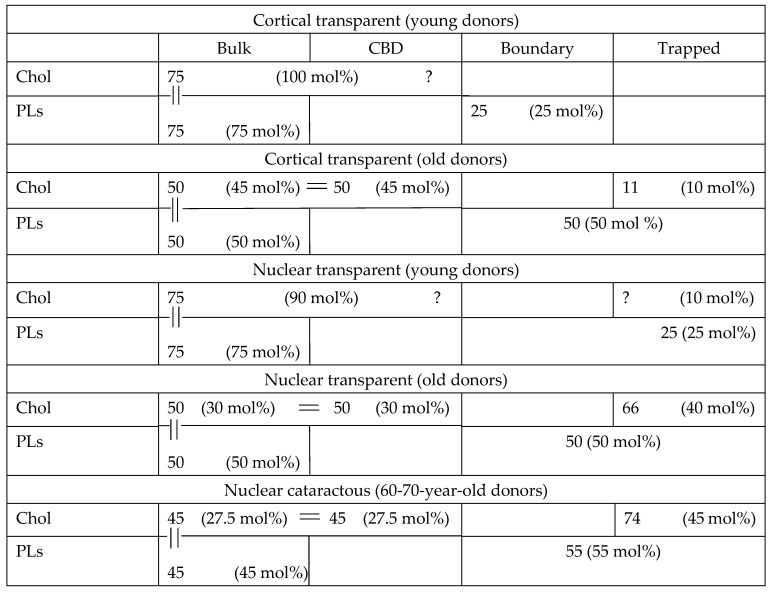

Numbers in parentheses indicate experimentally obtained relative amounts of Chol or PLs (mol% of total Chol or total PLs). To better understand distributions and connect distributions for Chol and PLs, we assume that, for all cases, we have 100 PL molecules in each membrane distributed according to the data obtained (i.e., numbers without parentheses). With the constraint indicated above that for all fiber cells the Chol/PL = 1 in bulk domains (indicated in the table as “||”), we can conclude that the number of Chol and PL molecules in the bulk domain is the same. This connects the distribution of numbers of Chol and PL molecules only in bulk domains. Another constraint indicated by “=” (for old cortical and nuclear membranes with Chol content above the Chol solubility threshold at Chol/PL = 2/1) enables calculation of the numbers of Chol molecules in other membrane domains (according to the measured distributions expressed in mol%). Some boxes (e.g., Chol in the bulk domain and CBD, as well as PLs in boundary and trapped lipids) combine the obtained data, which cannot be experimentally separated. Some boxes are empty because the method we used cannot evaluate the amounts of lipids in these domains (e.g., CBD for PLs and boundary for Chol) or because the measured results show that this type of lipid molecule cannot be incorporated in these domains (e.g., trapped Chol and trapped PLs). Question marks “?” indicate that the number of molecules cannot be evaluated with the existing data and the indicated constraints. The methodology should be developed further if we want to quantitatively determine PL and Chol content in each of the listed domains.

## Data Availability

Not applicable.
